# Jaundice and its associated factors among neonates admitted to selected referral hospitals in southwest oromia, Ethiopia: Multi-center cross-sectional study

**DOI:** 10.1016/j.heliyon.2023.e16019

**Published:** 2023-05-04

**Authors:** Gutu Belay, Asfaw Gerbi, Teka Gebremariam, Tsion Tilahun, Emebet Chimdi, Tesema Etefa

**Affiliations:** a Department of Medical Sciences, College of Medical and Health Sciences, Ambo University, Ethiopia; b Department of Biomedical Sciences (Anatomy), College of Medical Sciences, Institute of Health, Jimma University, Ethiopia; c Department of Pediatrics and Child Health, College of Medical Sciences, Institute of Health, Jimma University, Ethiopia

**Keywords:** Neonatal jaundice, Neonates, Factors, Ethiopia

## Abstract

**Background:**

Jaundice is a common clinical problem during the first month of birth throughout the world. Mainly, it is the leading cause of neonatal morbidity and mortality in developing countries.

**Objectives:**

The aimed of this studied was to assess predictors of jaundice among neonates admitted to selected referral hospitals in southwest Oromia, Ethiopia, 2021.

**Methods:**

An Institutional based cross-sectional study was implemented among 205 admitted neonates at selected referral hospitals in southwest Oromia, Ethiopia from October 05 to November 5, 2021. Jimma medical center (JMC), Wollega University referral hospital (WURH), and Ambo University Referral hospital (AURH) were selected by simple random sampling technique. A pretested structured interviewer-administered questionnaire and medical record review was used to collect data. Both binary and multivariable logistic regression analyses were performed to identify factors associated with neonatal jaundice. Logistic regression analyses were performed to identify factors associated with neonatal jaundice. Statistical Significance was declared at *P*-value less than 0.05 in the final model, and if the confidence interval does not include the null hypothesis value.

**Results:**

The prevalence of neonatal jaundice was 20.5% (95%CI: 1.74–1.85). The mean age of neonates was 8.6 ± 7.8 days. Traditional medicine use during current pregnancy (AOR: 5.62, 95%CI: 1.07, 9.52), Rh incompatibility (AOR: 0.045, 95%CI: 0.01, 0.21), gestational age (AOR: 4.61, 95%CI: 1.05, 10.3), premature rupture of membrane (AOR: 3.76, 95%CI: 1.58, 8.93) and hypertension (mother) (AOR: 3.99, 95%CI: 1.13, 14.02) were factors significantly associated with neonatal jaundice.

**Conclusion:**

Neonatal jaundice was relatively higher in the current study. Traditional medicine use, Rh incompatibility, premature ruptures of membrane, hypertension, and preterm gestational age were factors associated with neonatal jaundice.

## 1 Introduction

### 1.1 Background

Neonatal jaundice is the most common reason for hospitalization in the first week of life all over the world [1]. The term neonatal jaundice refers to the yellowish color of a newborn's skin and other membranes, which indicates high levels of unconjugated bilirubin in their blood [2,3].

In the first week following birth, 60% of term and 80% of preterm neonates experience clinical jaundice [4]. Physiological jaundice appears within 24 h of birth and can disappear on its own or worsen. Pathological jaundice, on the other hand, develops during the first 24 h of life [5]. According to estimations, 1.1 million babies worldwide may have severe hyperbilirubinemia each year, mostly occurring in Sub-Saharan Africa and South Asia [6]. Severe neonatal jaundice affects around 481,000 late-preterm and term newborn newborns worldwide every year, resulting in 114,000 deaths and over 63,000 survivors with long-term disabilities [7].

Sub-Saharan Africa shares the largest burden of neonatal jaundice-related to morbidity and mortality. The incidence of severe neonatal jaundice in these countries were 667.8 per 10,000 live birth [8]. In Ethiopia, more than the 34.5% of newborn death occurs within the first 28 days after birth [9]. Neonatal jaundice is one of the leading causes of neonatal mortality and morbidity, especially in developing countries including Ethiopia, and also the geographical variation might be contributed to the variation of its incidence among different continents [10,11].

As a result, neonatal jaundice is one of the leading causes of neonatal mortality and morbidity, particularly in developing countries such as Ethiopia, and geographical variation may play a role in the variation of its incidence across continents [12,13]. Pathological jaundice was one of the top ten leading causes of neonatal mortality, accounting for 7.5% of neonatal deaths, according to a retrospective study conducted in Jimma University Medical Center's (JUMC) neonatal intensive care unit (NICU) on Causes and Factors Associated with Neonatal Mortality in 2019 (14).

Severe neonatal jaundice results in acute bilirubin encephalopathy with a considerable risk of neonatal mortality and long-term neurologic complication. It is approximate 75% hospitalization which needs medical concern and hospital re-admission in the newborn [14]. A study conducted in developed countries indicated that blood compatibilities are the main reason for neonatal jaundice, but in developing countries prematurity, glucose 6-phosphate deficiency low birth weight, infection, and traditional practice were the main causes of neonatal jaundice [15]. The study indicated that elevation of total serum bilirubin level results in acute bilirubin encephalopathy that causes neurological problems. Infants surviving from jaundice may acquire long-term neurological complications such as sensor neural hearing loss, cerebral palsy, intellectual difficulties, and neurodevelopment delay later in life [16].

Even though the studies indicated pathological neonatal jaundice as top ten causes of neonatal death [17]. A limited study on the prevalence and risk factors of neonatal jaundice was conducted in the current study region. The study's findings are also helpful as planning and resource allocation information for policymakers and stakeholders. Furthermore, the findings of this study will form the basis for future clinical studies targeted at discovering causal linkages between neonatal jaundice and determinant factors.

## 2 Methods

### 2.1 Study area and period

The study was conducted in Southwest Oromia, Ethiopia from October 05 to November 5, 2021. Southwest Oromia has five referral hospitals. These are Jimma Medical Center (JMC), Wollega University referral hospital, Nekemte specialized hospital, Karl referral hospital, and Ambo University referral hospitals. Three hospitals Jimma Medical Center, Wollega University referral hospital, and Ambo University referral hospital were selected.

Jimma Medical Center (JMC) is located in Jimma town, Jimma zone, 355 km in South West of Addis Ababa, the capital city of Ethiopia. JMC is a teaching and referral hospital that gives general and specialized clinical services including maternal and childcare for about 20 million populations in the South-west part of Oromia. The neonatal intensive care unit is one of the wards in this hospital with over 25 beds where all neonates are referred from the JMC labor ward as well as from other nearby hospitals and health centers. The ward has 3 rooms for critically ill neonates and 5 other rooms for both neonates who are not severely ill and their mothers. JMC neonatal intensive care unit has been using locally made phototherapy since 2017 and advanced procedures such as exchange transfusions [18].

Wollega University referral hospital is located in Nekemte town, East Wollega Zone, 331 km in the West of Addis Ababa, the capital city of Ethiopia. The hospital catchment population is more than two million. The hospital has different wards like medical wards, gynecology and obstetrics ward, pediatrics ward including NICU and surgical ward, and has more than 33 beds.

Ambo university referral hospital is located in the Ambo town, West Shoa Zone, 114 km in the west of Addis Ababa. It has different wards including NICU which has 20 beds where all neonates referred from the labor ward and nearby health centers are admitted.

### 2.2 Study design

An institutional-based cross-sectional study design was employed at NICU at selected referral hospitals in southwest Oromia, Ethiopia.

### 2.3 Source population

All admitted neonates to NICU at referral hospitals in southwest Oromia and their mothers were our source population.

### 2.4 Study population

All randomly selected neonates admitted to NICU referral hospitals in southwest Oromia and their respective mothers who fulfilled inclusion criteria during the study period were our study population.

### 2.5 Eligibility criteria

All neonates admitted to NICU with age <28 days and their respective mothers were included in the study. The study excluded neonates with incomplete medical charts, extremely preterm neonates less than 28 weeks of gestational age, and neonates whose mothers were not present or deceased.

### 2.6 Sample size and sampling technique

The actual sample size was determined by using the single population proportion formula, where the following assumptions were considered, the prevalence of newborn jaundice was 37% (19) a study conducted in Mekelle, northern Ethiopia, with 95% confidence interval, and 5% margin of error. Because the overall population was 398, we used a correction formula and then added a 10% no response rate, yielding a final sample size of 208.

### 2.7 Sampling technique

Five referral hospitals are present in Southwest Oromia. Jimma medical center (JMC), Wollega University referral hospital (WURH), and Ambo University Referral hospital (AURH) were selected by simple random sampling technique. Total admissions of neonates in these three hospitals were 1644, 1620, and 1512 at JMC, WURH, and AURH respectively from the HMIS report in 2013E.C. From this monthly average number of neonates admitted to the neonatal intensive care unit at JMC, WURH, and AURH were 137, 135, and 126 respectively by dividing total admitted neonates into twelve months. By adding these average total number of neonates admitted in these three hospitals was 398 and was taken as a frame of reference.

The calculated sample size (208) was proportionally allocated by using a proportional allocation formula Where, the sample size for each hospital, n = a total sample size to be selected, N = total number of neonates admitted in three hospitals (398), and a total number of admitted neonates in each hospital. Accordingly, 72 neonates from JMC, 70 neonates from WURH, and 66 neonates from AURH were allocated.

The required number of individuals was selected by systematic random sampling method after calculating the interval (K) by dividing the total number of admitted neonates per month at each hospital to desired sample size for each hospital. Therefore, for JMC K = = 2, WURH K = = 2, and AURH K = = 2.

**Fig. 1:**
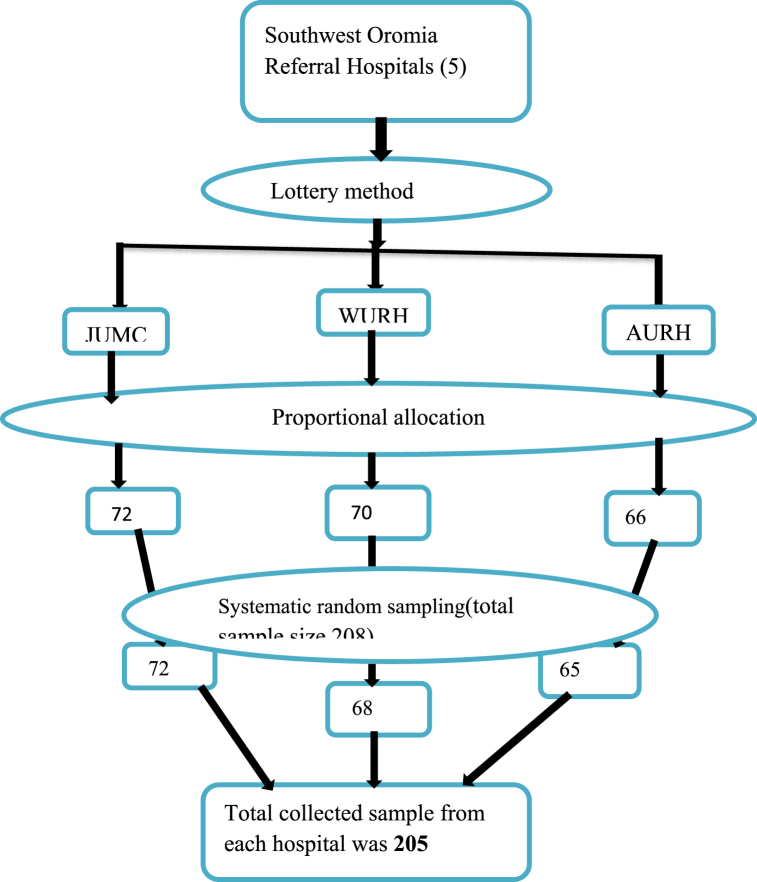
Schematic representation of sampling technique of admitted neonates at three referral hospitals in southwest Oromia, Ethiopia, 2021.

The first participant was selected by lottery method from the K participant of admission registration file of neonates admitted in NICU during the data collection period. Finally, data were collected from every K [2] individual from the randomly selected participants based on the admission sequence of the registration files, and neonates admitted during the data collection period were selected based on their case flow. If randomly selected neonates were not fulfilled inclusion criteria; the next neonates were selected. A total of 205 neonates were selected through the above procedure as indicated in Fig. 1.

### 2.8 Data collection tool and procedure

Data were collected face-to-face using interview-based structured questionnaires and medical record review. The data collection tools were adopted and modified from a review of different kinds of literature [[19], [20], [21], [22], [23], [24]]. The data collection tool has different sections such as socio-demographic factors, maternal characteristics, and neonatal factors for neonatal jaundice. Maternal obstetric and fetal related data that might not be addressed by interviews, such as oxytocin during labor, birth weight (kg), hypothermia, sepsis, Rh incompatibility, blood type incompatibility, mother's blood group, and neonate's blood group were collected from patient medical records.

Neonatal jaundice was collected from the medical chart after being diagnosed by the physician. The time onset of jaundice was collected from the mothers and medical chart for confirmation to classify jaundice. Using the time onset; jaundice was classified as pathological and physiological jaundice. Jaundice onset within 24 h births was classified as pathological and onset after 24 h was physiological jaundice [25].

### 2.9 Operational definition and definition of terms

• **Preterm:** neonate born less than 37wks gestational age
• **Extremely preterm**: neonates less than 28 weeks gestational age [26].
• **Low birth weight**: Neonate with birth weight less than 2.50 kg
• **Neonatal jaundice**: Neonates diagnosed as jaundiced by the physician
• **Pathological jaundice:** Jaundice visible within 24 h s and persistent for more than one week in term (bilirubin >12 mg/dl) and more than 2 weeks in preterm (bilirubin level >15 mg/dl) [27].
• **Premature rupture of membrane (PROM):** Rupture of the fetal membranes after the 28th week of GA and before the onset of labor.
• **Birth injuries**: Physical injuries experienced during childbirth, and can affect either the mother or the baby.
• **Critically ill:** Mother is unable to respond to questions because she is suffering from a life-threatening multisystem condition that can result in substantial morbidity or mortality.
• **Traditional medicine use:** described as any culturally used medication by mothers during her pregnancy for any purpose and not prescribed by health professionals.

### 2.10 Data processing and analysis

Data from three hospitals were checked for completeness, cleaned, and entered using Epi Data-Version 0.3.1 and exported to SPSS version 25 for analysis. To compare the prevalence of neonatal jaundice in three hospitals data were separately treated. For descriptive statistics and logistic regression, the data were mixed up and analyzed. Descriptive statistics were used to summarize the data using frequencies, percentages, and graphs. Both binary and multivariable logistic regression analyses were performed to identify factors associated with neonatal jaundice. The goodness of fit test designed by Hosmer and Lameshow was used to assess model fitness. The Hosmer-Lameshow goodness of fit test was used to determine model fitness, and the model was found to be properly fit with a p > 0.05. The variables in bi-variable analysis with p < 0.25 were entered into a multivariable logistic regression model. AOR with a 95% CI was used to determine the association between dependent and independent variables. The statistical significance of the association between dependent and independent variables was declared at p-value <0.05, and also statistically significant if the confidence interval does not include the null hypothesis value.

### 2.11 Data quality management

To assure the quality of data, clarity, and understandability a pretest was done on 5% of the sample size at Shambu general hospital, and necessary modifications were done. The data collection tool prepared in English was translated to Afaan Oromo and Amharic and then retranslated back to English to check its consistency. Data were collected by fluent speakers’ local languages under regular supervision. Regular daily supervision was done for checking the consistency and completeness of the questionnaires on the daily basis by the supervisors.

## 3 Result

### 3.1 Socio demographic characteristics of respondents

A total of 205 newborns were enrolled in the study, with a 100% response rate for those who had complete information on their laboratory records and medical record review chart.

From maternal socio-demographic characteristics of respondents, most mothers 142 (69.3%) were between 20 and 34 years with a mean age of 26 + 5.5 y.rs. More than half 116 (56.6%) of mothers were from rural areas. About two-thirds 150 (73.2%) of respondents were unemployed (as shown in Table 1).

**Table 1:** Maternal socio-demographic characteristics of respondents at three referral hospitals in southwest Oromia, Ethiopia, 2021.

Variables	Category	Frequency (n = 205)	Percent (%)
Age of mothers (years)	<20	37	18.0%
	20–34	142	69.3%
	≥35	26	12.7%
Residence	Urban	89	43.4%
	Rural	116	56.6%
Educational level	Illiterate	69	33.7%
	Primary school	72	35.1%
	Secondary school	45	22.0%
	College and above	19	9.3%
Maternal	Gov. Employed	49	23.9%
Occupation	unemployed	156	76.1%

### 3.2 Maternal obstetric characteristics

From obstetric characteristics of respondents, the majority of 191 (92.7%) of mothers were followed antenatal care. More than half 112 (54.6%) of mothers were the Primiparous. About 109 (53.2%) were prim gravida. Below ten percent 12 (5.9%) of mothers were taking traditional medicine. Nearly 3 (1.5%) of mothers had a history of blood transfusion during pregnancy. About 10 (4.9%) of mothers were delivered at home. Less than one-third 33 (16.1%) of mothers were used oxytocin for induction during labor. Mothers who had a history of premature rupture of the membrane were accounted for 39 (19%). The majority of mothers’ blood groups were A+ and Rh-positive which was 76 (37.1%) and 186 (90.7%) respectively. Less than ten percent 13 (6.3%) of mothers were having a history of gestational diabetic Mellitus whereas 14 (6.8%) mothers were a history of gestational hypertension (as shown in Table 2).

**Table 2:** Obstetric characteristics of respondents at three referral hospitals in southwest Oromia, Ethiopia, 2021.

Variables	Category	Frequency (n = 205)	Percent (%)
Have ANC follow up	Yes	191	92.7%
	No	14	7.3%
Parity	Prim para	112	54.6%
	Multipara	93	45.4%
Gravidity	Prim gravida	109	53.2%
	Multigravida	96	46.8%
Traditional medicine take	Yes	12	5.9%
	No	193	94.1%
Blood transfusion	Yes	3	1.5%
	No	202	98.5%
Place of delivery	Home	10	4.9%
	Health institution	195	95.1%
Mode of delivery	SVD	171	83.4%
	Cesarean section	20	9.8%
	Instrumental	14	6.8%
Oxytocin use for induction	No	172	83.9%
	Yes	33	16.1%
Time of delivery	Day	116	56.6%
	Night	89	43.4%
DOL	Normal	172	83.9%
	Prolonged	33	16.1%
PROM history	Yes	39	19.0%
	No	166	81.0%
Mothers blood group	A	76	37.10%
	B	41	20.00%
	AB	20	9.80%
	O	31	15.10%
	O-	14	6.80%
	Unknown	23	11.20%
Mother Rh status	Positive	186	90.7%
	Negative	19	9.3%
Diabetic mellitus	No	13	6.3%
	Yes	192	93.7%
Hypertension	Yes	14	6.8%
	No	191	93.2%

DOL-duration of labor, PROM-premature ruptures of membrane, SVD-spontaneous vaginal delivery, ANC- Antenatal care.

### 3.3 Neonatal characteristics

The mean age of neonates was 8.6 ± 7.8 days. Most of the neonates were male 127 (62%). The birth weight of neonates was more than half 112 (54.6%) were normal. About 24 (11.7%) of neonates had ABO incompatibility whereas 17 (8.3%) had Rh incompatibility. Only 11 (5.4%) have a history of sibling jaundice. More than half 113 (55.1%) of neonates had neonatal sepsis and almost half 106 (51.7%) of them had hypothermia. Only 13 (6.3%) of neonates had birth trauma. Around 80 (39%) of neonates were not breastfed (as shown in Table 3).

**Table 3:** Characteristics admitted neonates at three referral hospitals in southwest Oromia, Ethiopia, 2021.

Variables	Category	Frequency (n = 205)	Percent (%)
Age of neonates	<2 days	28	13.7%
	2–7days	87	42.4%
	≥8 days	90	43.9%
Sex of neonates	Male	127	62.0%
	Female	78	38.0%
Birth weight	<2500 g	68	33.2%
	2500 g m-3500 g	112	54.6%
	>3500 g	25	12.2%
Gestational age	Term	135	65.9%
	Preterm	55	26.8%
	Post-term	15	7.3%
Neonates blood group	A	73	35.6%
	B	53	25.9%
	AB	24	11.7%
	O	37	18.4%
	O-	18	8.8%
ABO incompatibility	No	181	88.3%
	Yes	24	11.7%
Rh incompatibility	Yes	17	8.3%
	No	188	91.7%
Sibling jaundice	No	194	94.6%
	Yes	11	5.4%
sepsis	Yes	113	55.1%
	No	92	44.9%
Hypothermia	Yes	106	51.7%
	No	101	49.3%
Birth trauma	No	192	93.7%
	Yes	13	6.3%
Breast feed	Yes	125	61.0%
	No	80	39.0%

### 3.4 Prevalence of neonatal jaundice

From 42 ((20.5%) 95%CI: 1.74–1.85) jaundiced newborns, approximately 32 (15.6%) developed within the first 24 h after delivery and 10 (4.9%) developed after the first 24 h. Therefore, about 15.6% were pathological jaundice and 4.9% were physiological jaundice. Only 3 (1.5%) had acute bilirubin encephalopathy.

### 3.5 Independent predictors of neonatal jaundice

To identify the association between neonatal jaundice and predictive variables, bi-variable logistic regression analysis was first done for all independent variables. A total of seventeen variables were found to be associated in the crude analysis were a candidate for multivariable analysis with a p-value <0.25. Five variables, namely: traditional medicine use during pregnancy, Rh incompatibility, gestational age, premature rupture of membrane, and hypertension showed significant association with neonatal jaundice among neonates.

Neonates born to mothers who took unknown traditional medicine during pregnancy were 5.62 times more likely to have neonatal jaundice than those who were not taken traditional medicine (AOR = 5.62 95% CI: 1.07, 9.52). Neonates who were born from mothers who had a history of PROM were 3.76 times more likely to have neonatal jaundice than neonates born from mothers without PROM history (AOR = 3.76 95% CI: 1.58,8.93). Neonates who were born from hypertensive mothers were 3.99 times more likely to have neonatal jaundice as compared with neonates born to non-hypertensive mothers (AOR = 3.99 95% CI: 1.13,14.02). The risk of developing neonatal jaundice was 4.61 (95% CI: 1.05, 10.3′) times higher with preterm neonates as compared to term neonates. Neonates who had no Rh incompatibility were 95.5% less likely to have neonatal jaundice as compared to neonates with Rh incompatibility (as shown in Table 4).

**Table 4:** Bi-variable and multivariable analysis factors associated with neonatal jaundice among admitted neonates at three referral hospitals in southwest Oromia, Ethiopia, 2021.

Variable	Category	Jaundice		Bi-variable analysis		Multivariable analysis	
		Yes	No	COR (95% CI)	p-value	AOR (95% C·I)	p-value
Residence	Urban	22	67	1		1	
	Rural	20	96	1.57 (0.32, 1.25)	0.191	0.73 (0.28, 1.93)	0.537
Maternal	Employed	7	42	1		1	
Occupation	Unemployed	35	121	0.57 (0.24, 1.39)	0.222	1.51 (0.44, 5.16)	0.511
ANC use	Yes	36	154	1		1	
	No	6	9	0.35 (0.12, 1.04)	0.061	3.61 (0.68, 19.02)	0.129
Traditional Medici use	No	37	156	1		1	
	Yes	5	7	0.33 (0.10, 1.10)	0.072	5.62 (1.07, 9.52)	0.041^a^
Place of delivery	HI	37	158	1		1	
	Home	5	5	0.23 (0.06, 0.85)	0.027	4.38 (0.78, 16.71)	0.094
Mode of delivery	SVD	31	140	1		1	
	C/S	6	14	0.52 (0.18, 1.45)	0.210	3.30 (0.49, 21.92)	0.051
	Instrument	5	9	0.39 (0.12, 1.27)	0.120	3.23 (0.33,31.02)	0.320
oxytocin use	No	30	81	1		1	
	Yes	12	82	2.53 (1.21,5.28)	0.013	0.32 (0.101, 1.01)	0.052
PROM history	No	27	139	1		1	
	Yes	15	24	0.31 (0.14,0.66)	0.003	3.76 (1.58,8.93)	0.003^a^
Diabetic mellitus	No	37	155	1		1	
	Yes	5	8	0.38 (0.11, 2.34)	0.108	0.90 (0.103, 7.87)	0.925
Hypertension	No	34	157	1		1	
	Yes	8	6	0.16 (0.05,0 .49)	0.001	3.99 (1.13,14.02)	0.031^a^
Gestational age	Term	14	121	1		1	
	Preterm	22	33	0.17 (0.08, 0.37)	0.000	4.61 (1.05, 10.3)	0.043^a^
	Post term	6	9	0.17 (0.05, 0.56)	0.003	1.66 (0.29, 9.30)	0.564
Birth weight	NBW	14	98	1		1	
	LBW	23	45	0.28 (0.13, 0.59)	0.001	4.17 (0.89, 19.49)	0.070
	HBW	5	20	0.57 (0.18, 1.76)	0.331	2.29 (0.48, 10.87)	0.296
Neonates blood group	A	11	62	1		1	
	B	10	43	0.76 (0.29, 1.95)	0.572	1.36 (0.29, 6.29)	0.692
	AB	6	18	0.53 (0.17, 1.63)	0.272	1.93 (0.40, 9.29)	0.412
	O	10	27	0.47 (0.18, 1.26)	0.136	1.46 (0.20, 10.58)	0.704
	O-	5	13	0.46 (0.13, 1.55)	0.212	1.78 (0.32, 9.75)	0.505
ABO incompatibility	No	34	147	1		1	
	Yes	8	16	0.46 (0.18, 1.16)	0.103	1.51 (0.33, 6.83)	0.592
Rh incompatibility	Yes	11	6	1		1	
	No	31	157	9.28 (3.9,26.98)	0.000	0.045 (0.01, 0.21)	0.000^a^
Sibling jaundice	No	36	158	1		1	
	Yes	6	5	0.19 (0.05, 0.65)	0.009	2.25 (0.39, 12.83)	0.361
Birth trauma	No	36	156	1		1	
	Yes	6	7	0.26 (0.08, 0.84)	0.025	3.01 (0.59, 15.11)	0.182

a Value statistically significant (*P*-value< 0.05) COR- Crude odds ratio AOR- Adjusted Odds ratio CI-Confidence interval 1- reference.

## 4 Discussion

From total of 205 neonates participated in the current study, the prevalence of neonatal jaundice were 42 (20.5%) (95%CI: 1.74–1.85). This finding was consistent with previous studies conducted at Nambya in Uganda (22.7%) [28].

The observed finding was lower than studies done in, Bloemfontein (55.2%) [29], Rwanda (44.3%) [30], Nigeria (32.6%) [31], Nepal (39.95%) [32], and Mekelle Northern Ethiopia (37.3%) [33]. Possible reason for lower prevalence observed compared to in Malaysia, Nepal and Nigeria was due to difference in methodology, sample size and study population. The variability of current study from Rwanda study conducted in Rwanda was retrospective over two years of admitted neonates. The variation between current study and Mekelle Ethiopia might be the current study was done only at referral hospitals where more obstetric care is expected and study period for Mekelle was three month whereas the current was only one month study period.

The current study was higher than study done in Congo (7.2%) [34], and Nairobi Kenya (16%) [35]. Possible reason might be a methodological variation, and if bilirubin level 5 mg/dl and exclude the clinical jaundiced with low bilirubin level as compared to our study setting. The reason for Windhoek Namibia might be methodological variation that study in Namibia excludes neonates with hemolysis, unknown date of birth, delayed in transient and being icteric (yellow sclera and skin). The difference for Brazzaville Congo might be Study setting in one hospital and they includes only if bilirubin level 5 mg/dl and excluding the clinical jaundiced with low bilirubin level comparatively the current study includes both clinical jaundiced with low bilirubin and high level of bilirubin as jaundiced.

Traditional medicine use during pregnancy, Rh incompatibility, and preterm gestational age, premature rupture of membrane and hypertension during pregnancy were factors associated with neonatal jaundice. Rh incompatibility was found to be significantly associated with neonatal jaundice. This finding was in line with study done in Turkey on factors affecting neonatal jaundice 80 (11.3%) of neonatal jaundice was Rh incompatibility [36]. In addition, this study was in line with previous studies conducted in Mekelle Northern Ethiopia [37]. The possible explanation may be Rh incompatibility between maternal and fetal cause hemolytic. This develop when mother Rh negative and fetus Rh positive that results in formation of antibodies in maternal blood which attack fetal red blood cells. The fetal red blood cells broken down can develop hyperbilirubinemia [38].

According to our study, unknown traditional medicine use during pregnancy was significantly associated with neonatal jaundice. This finding was in line with the studies conducted in Malaysia [39], and Nigeria [40]. The possible explanation could be traditional medicine used during pregnancy could cross the placenta and affect fetal liver that might result in decreased bilirubin clearance from the body and cause hyperbilirubinemia [41,42]. Medicinal plants and herbal remedies contain substances that can be toxic to the human body and the fetus. Potential effects of indiscriminate use of medicinal plants are embryotoxicity, teratogenic, and abortifacient effects [43].

Preterm gestational age was significantly associated with neonatal jaundice. This finding was in line with studies conducted in Iran [44,45], Rwanda [30], Nigeria [46], Benin Nigeria [47] and Denmark [48]. The possible cause might be due to short life span of erythrocyte in premature neonates result in increased bilirubin production and reduced bilirubin elimination capacity [49].

According to our study, premature rupture of membrane was significantly associated with neonatal jaundice. Our finding was compatible with studies done in Turkey [36], Iran [50], and Indian [51]. The possible explanation might be premature rupture of membrane results in preterm delivery which result in immaturity of liver that cannot get rid of much bilirubin from the body [52].

According to the current study, gestational hypertension was significantly associated with neonatal jaundice. This finding was in line with studies done in Turkey [36], India [53], Iran [54] and China [55]. The possible reason is hypertension during pregnancy leads to cause abnormal coagulation profile with bleeding on babies which increase bilirubin level in the blood [56]. In addition, gestational hypertension might be result in prematurity which fails to conjugate normally produced bilirubin from red blood cell which results in jaundice [57].

### 4.1 Strength of the study

A study was conducted at multicenter cross-sectional study. The study identifies new factors associated with outcome. The study is important for researchers as bassline to identify definite etiologies and underlying causes of neonatal jaundice.

### 4.2 Limitation of the study

As the study design was cross-sectional; it is difficult to form the causal relationship between neonatal jaundice and the associated factors. Due to small sample was obtained from each hospital; this study didn't compare among three hospitals regarding risk factors of neonatal jaundice.

## 5 Conclusion

The prevalence of neonatal jaundice in southwest Oromia referral hospitals was relatively higher. Preterm gestational age at birth Rh incompatibility, premature rupture of membrane, use of unknown traditional medicine during pregnancy, and gestational hypertension were factors significantly associated with neonatal jaundice. For Researchers, longitudinal studies should be important to identify definite etiologies and underlying causes of neonatal jaundice.

## Ethical statement

The study was conducted after ethical approval from Research Ethical Committee of Jimma University with IHR/554/21. After permission secured from the hospital data collection was started. Written, informed consent was obtained from all participants by the local language prior to interview. Data was kept confidential. The rights to withdraw from the study were respected for all.

## Informed consent

Informed consent was obtained from all the participants included in the study.

## Author contribution statement

Gutu Belay: Conceived and designed the experiments; Performed the experiments; Analyzed and interpreted the data; Contributed reagents, materials, analysis tools or data; Wrote the paper.

Asfaw Gerbi; Tesema Etefa: Conceived and designed the experiments; Performed the experiments; Wrote the paper.

Teka Gebremariam: Performed the experiments; Analyzed and interpreted the data.

Tsion Tilahun: Conceived and designed the experiments; Contributed reagents, materials, analysis tools or data; Wrote the paper.

Emebet Chimdi: Analyzed and interpreted the data; Contributed reagents, materials, analysis tools or data.

## Data availability statement

Data included in article/supplementary material/referenced in article.

## Declaration of competing interest

The authors declare no conflict of interest.

## Abbreviations

AURH: Ambo University Referral Hospital
CI: confidence interval
HMIS: health management information system
JMC: Jimma Medical Center
NICU: neonatal intensive care unit
OR: odds ratio
PROM: Premature rupture of membrane
SVD: save vaginal delivery
WURH: Wollega University Referral Hospital
